# Sleep spindles are linked to white matter connectivity differentially in older adults with and without cognitive impairment

**DOI:** 10.1093/braincomms/fcag303

**Published:** 2026-08-10

**Authors:** Aaron Lam, Shawn Dexiao Kong, Jake R Palmer, Fernando Calamante, Hannes Almgren, Angela L D’Rozario, Sharon L Naismith

**Affiliations:** School of Psychology, The University of Sydney, Sydney, NSW 2050, Australia; Brain and Mind Centre and Charles Perkins Centre, The University of Sydney, Sydney 2050, NSW, Australia; CIRUS, Sleep and Circadian Research Group, Woolcock Institute of Medical Research, Macquarie University, Sydney 2113, NSW, Australia; School of Psychology, The University of Sydney, Sydney, NSW 2050, Australia; Brain and Mind Centre and Charles Perkins Centre, The University of Sydney, Sydney 2050, NSW, Australia; School of Psychology, The University of Sydney, Sydney, NSW 2050, Australia; Brain and Mind Centre and Charles Perkins Centre, The University of Sydney, Sydney 2050, NSW, Australia; Sydney Imaging and School of Biomedical Engineering, The University of Sydney, Sydney 2050, NSW, Australia; Sydney Imaging and School of Biomedical Engineering, The University of Sydney, Sydney 2050, NSW, Australia; School of Psychology, The University of Sydney, Sydney, NSW 2050, Australia; CIRUS, Sleep and Circadian Research Group, Woolcock Institute of Medical Research, Macquarie University, Sydney 2113, NSW, Australia; School of Psychological Sciences, Macquarie University, Sydney 2113, NSW, Australia; School of Psychology, The University of Sydney, Sydney, NSW 2050, Australia; Brain and Mind Centre and Charles Perkins Centre, The University of Sydney, Sydney 2050, NSW, Australia

**Keywords:** dementia, ageing, memory, neuroimaging, slow oscillations

## Abstract

While sleep disturbance increases with age and neurodegeneration, the relationship between sleep micro-architecture and changes in key white matter tracts is unknown. We aimed to determine how sleep spindles and slow oscillations relate to structural connectivity within selected white matter tracts in older adults at risk for dementia. In this cross-sectional study, 67 participants (mean age = 68.1; 47 females) with subjective cognitive decline (*n* = 21) or mild cognitive impairment (*n* = 46) underwent neuropsychological assessment, magnetic resonance imaging and polysomnography. White matter connectivity of anterior thalamic radiation and superior longitudinal fasciculus subdivisions II and III was quantified using whole-brain tractography, with left and right tracts combined. Primary sleep micro-architecture outcomes included slow (11–13 Hz) and fast (13–16 Hz) spindle density and slow oscillation (0.25–1 Hz) power. Secondary outcomes included: overall spindle density (11–16 Hz) and amplitude. Linear regressions assessed associations between sleep micro-architecture and structural connectivity, adjusting for age and sex, with cognitive classification as a moderator. Multiple comparisons were controlled for using the Benjamini–Hochberg false discovery rate procedure, with *q* < 0.1 considered statistically significant. The mild cognitive impairment group exhibited lower frontal and central fast spindle density compared to the subjective cognitive impairment group (*P* < 0.05), with no other group differences in sleep macro-architecture or slow oscillatory activity. After false discovery rate correction, there were no significant associations between slow spindle density, fast spindle density or slow oscillation power and tract connectivity. An interaction between central fast spindle density and superior longitudinal fasciculus III connectivity was observed prior to false discovery rate correction, explaining 8% of unique variance, but did not remain significant after correction (*q* > 0.10). Exploratory analyses reveal a significant interaction between superior longitudinal fasciculus II connectivity and cognitive classification on spindle amplitude, with greater connectivity associated with higher spindle amplitude in individuals with mild cognitive impairment (*P* = 0.001), but not in those with subjective cognitive decline (*P* > 0.05). Overall, white matter connectivity of the anterior thalamic radiation and superior longitudinal fasciculus was not robustly associated with sleep spindle density or slow oscillatory power in this at-risk cohort. However, superior longitudinal fasciculus II connectivity may differentially relate to spindle amplitude in mild cognitive impairment, potentially reflecting altered thalamocortical network engagement in early neurodegeneration.

## Teaser

Lam *et al*. report that specific white matter pathways support sleep rhythms important for memory. Fast sleep spindles relate to anterior thalamic connectivity, while broader frontoparietal connections are linked to larger spindles in mild cognitive impairment, suggesting early compensatory network recruitment during ageing and neurodegeneration.

## Graphical Abstract

**Graphical Abstract:**
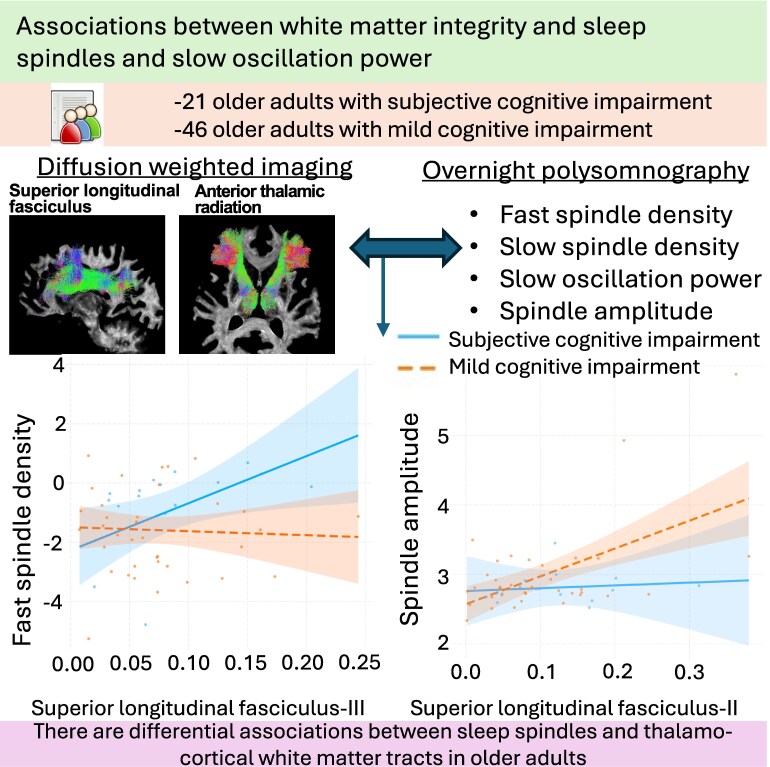

## Introduction

Sleep changes have emerged as an important feature of ageing.^1^ Alterations in sleep macro-architecture in older adults are well established,^2^ and include advanced sleep phase,^3^ reduced total sleep time, greater nocturnal awakenings and poorer sleep efficiency.^4-6^ Significantly, these sleep disturbances have been associated with greater risk of incident Alzheimer’s disease (Ad), vascular dementia and all-cause dementia.^7^ Understanding the neurobiological underpinnings of these alterations in individuals at risk for, or experiencing, cognitive decline will be critical for directing development of targeted interventions aimed at improving both sleep quality and cognitive functioning.

Human sleep micro-architecture consists of temporally synchronized neural oscillations that occur in distinct brain networks and during particular sleep stages.^8^ In the ageing literature, sleep spindles and slow wave activity during NREM sleep have attracted particular interest. Spindles are characterized by distinct periods of waxing and waning of electroencephalography (EEG) oscillations during NREM sleep (11.0 Hz–16.0 Hz range) ^9^ that are generated in specialized cells of the thalamic reticular nucleus, and propagate via thalamo-cortical brain networks.^10^ Within the sleep spindle range, slow spindles (11.0 Hz–13.0 Hz range) predominate over frontal regions and fast spindles (13.0 Hz–16.0 Hz range) over centro-parietal areas.^11^

Spindle activity is well established as a critical component of cortical synaptic plasticity^9^ and overnight memory consolidation,^12-14^ with reductions in spindles observed in individuals with mild cognitive impairment (MCI) and Ad.^3,15-17^ Experimental evidence suggests the temporal synchronization of spindles and slow-wave oscillations is impaired in older adults, which subsequently impairs overnight memory consolidation.^18^ Furthermore, spindles have been shown to mediate the relationship between ptau181 and cognition, as well as predict cognitive performance over time in older adults with AD.^19^ While the cognitive implications of impaired slow oscillations have been a significant focus of the field, recent evidence suggests slow oscillations may actually play a critical role in glymphatic clearance and, subsequently, a causal role in the pathophysiology of AD.^20^ Slow oscillations have been directly linked to Ad pathology. For instance, one study^21^ showed the proportion of slow oscillatory activity (between 0.6 and 1 Hz) to be predictive of future A*β* deposition in cognitive normal older adults (*n* = 32), beyond the effects of age, sex and sleep apnoea. In addition, the decline in slow-wave sleep has been shown to be accelerated in Ad (see review^1^).

Previous efforts to elucidate the neurobiological features underpinning these neural oscillations during sleep in healthy and pathological ageing have primarily focused on cortical grey matter changes^18,22^ or subcortical grey matter structures.^23^ However, white matter alterations are another well-established feature of ageing that is also more pronounced in individuals at-risk for dementia.^24^ Given that neural oscillations during sleep propagate over multiple brain regions in highly synchronized patterns, it is plausible that white matter damage may disrupt or reduce the power of these signals. Despite this, there is a paucity of literature investigating the relationship between white matter and sleep micro-architecture in older adults generally and in those with prodromal forms of dementia specifically.

Two prior studies have investigated associations between sleep spindles and white matter micro-structure in healthy younger and older adults. In a sample of 30 young and 31 older participants, white matter micro-structure derived from diffusion tensor imaging was related to spindle amplitude in the younger group only, with effects found primarily in the anterior thalamic radiation (ATR), superior longitudinal fasciculus (SLF), inferior fronto-occipital fasciculus and forceps minor.^25^ Another study found that spindle density during the fourth quarter of NREM sleep was associated with motor learning consolidation in both healthy younger (*n* = 40) and older (*n* = 51) adults.^26^ This effect was moderated by white matter micro-structure in a number of regions containing white matter pathways overlapping those identified by the previous study^25^ including cortical association fibres such as the SLF and cortico-subcortical connections such as the ATR. On the other hand, slow oscillations are generated primarily within the prefrontal cortex and propagate across widespread cortical regions as travelling waves during NREM sleep.^27^ The propagation of these waves may rely on coordinated activity from large-scale cortical networks. The SLF is a major long-range association connecting frontal and parietal cortices. Given that slow oscillations typically originate in frontal regions and propagate posteriorly towards parietal cortex, structural integrity of frontoparietal white matter pathways such as the SLF may be critical. There are no known studies that examine relationships between white matter and sleep micro-architecture in older adults at risk for dementia.

While the approach used to assess white matter micro-structural properties used in the above studies provides insight into local white matter alterations, it lacks anatomical accuracy^28^ and is limited by the use of the diffusion tensor model, which has been shown to be insufficient for modelling regions with more than one fibre orientation.^29,30^ Further, local white matter properties only indirectly represent the ability of a white matter fibre bundle to transmit information between its endpoints, often referred to as white matter connectivity. However, white matter connectivity may represent a critical element supporting sleep micro-architecture given sleep spindles and slow-wave oscillations are dynamic spatio-temporal processes that propagate in specific patterns and throughout distinct brain networks,^16,31^ and are therefore likely to be reliant on efficient and effective brain connectivity.

Probabilistic tractography based on modern diffusion models, such as spherical deconvolution,^32^ combined with state-of-the-art tractography optimization methods^30,33^ now provides the opportunity to quantify the connectivity of specific white matter fibre bundles. One such metric that addresses previous limitations is fibre bundle capacity (FBC), which aims to quantify the potential of a fibre bundle to transmit information.^34^ In contrast with other widely used diffusion-based metrics that characterize *local* diffusion properties within a voxel, FBC is derived from tractography streamlines and quantifies the total intra-axonal cross-sectional area of connections within an entire white matter fibre bundle of interest. Importantly, this measure thus allows biological interpretations to be drawn that may not be possible with other tractography-based measures.

Despite growing evidence linking sleep disturbances and white matter degeneration in ageing and neurodegeneration, no studies to date have examined the relationship between sleep micro-architecture and white matter connectivity in older adults at risk for dementia. The current study aimed to determine, in older adults at risk of dementia, the extent to which fast and slow spindle density and slow oscillation (<1 Hz) EEG power during NREM sleep is related to white matter connectivity of selected white matter fibre bundles—the ATR, SLF-II and SLF-III were selected as key tracts of interest based on previous studies^25,26^ and their potential involvement in propagating neural oscillations from the thalamus and throughout the cortex. We hypothesized that increased spindle density would be related to greater connectivity of each tract, while increased slow oscillatory power would be preferentially associated with the greater cortico-cortico connectivity of the SLF. Given overall (fast + slow) spindle density and spindle amplitude have previously been assessed in the literature ^25,26^ we included these measures as exploratory outcomes.

## Materials and methods

### Participants

Participants were recruited from the Healthy Brain Ageing Clinic at the Brain and Mind Centre, University of Sydney, a specialist memory and cognition research clinic for individuals with new onset concerns about their mood or cognition. Inclusion requires individuals to be aged over 50 years, to have a Mini-Mental State Examination (MMSE) > 20 and to have a referral for assessment from their general practitioner or specialist. Exclusion criteria are age less than 50 years, primary language other than English; significant neurological disorder (e.g. epilepsy, Parkinson’s disease): history of significant psychiatric illness (e.g. psychosis, bipolar disorder); previous stroke or head injury with loss of consciousness exceeding 30 min; and, history of significant substance misuse. For this sub-study, additional inclusion criteria required participants to have undergone an MRI scan and overnight polysomnography (PSG) within three months and to have an MMSE ≥24. Participants were also not included in this analysis if they were taking medications under the following classes: hypnotics, sedatives, mood stabilizers, anti-epileptics, anti-psychotics, benzodiazepines and cholinesterase inhibitors. All participants provided written informed consent before participation in the study and all research activities were approved by the University of Sydney Human Research Ethics Committee.

### Clinical assessment

All participants underwent detailed medical, psychological and neuropsychological assessment as described previously.^35^ Medical assessment was completed by a geriatrician and was based on a semi-structured interview that gathered detailed medical history, medication use and assessment of medical burden (Cumulative Illness Rating Scale-Geriatric version^36^). Height and weight were also measured to derive body mass index. Participants additionally completed a series of self-report questionnaires to assess subjective sleep quality (Pittsburgh Sleep Quality Index^37^) and current depressive symptoms (Geriatric Depression Scale^38^).

While detailed neuropsychological assessment was also completed, we report the MMSE score for descriptive purposes as a measure of global cognition. Premorbid intelligence was estimated using the Wechsler Test of Adult Reading (WTAR).^39^ The WTAR is a standardized measure of word reading ability that provides an estimate of intellectual functioning prior to the onset of neurological decline. From these assessments, MCI classifications were made by consensus rating between the geriatrician and two neuropsychologists. Classifications were made according to established criteria requiring objective evidence of cognitive impairment without evidence of impairment in everyday functional abilities.^40^ Furthermore, this classification followed standard clinical criteria, whereby cognitive performance was interpreted relative to estimated premorbid functioning, educational and occupational attainment rather than solely against population normative z-scores. This approach enables detection of clinically meaningful decline in individuals with high premorbid ability.^41^ As all participants sought referral to the clinic for concerns regarding their cognition, those who did not meet criteria for MCI were classified as experiencing subjective cognitive decline (SCD).

### Overnight polysomnography

As previously described,^42^ participants completed overnight in-laboratory PSG. Participants maintained their usual bed and wake times for the sleep study and were asked to avoid caffeine, alcohol and daytime sleep on the day of the sleep study. The PSG montage used included EEG recorded at scalp locations Fz-average of M1 + M2, F3-M2, F4-M1, Cz-average of M1 + M2, C3-M2 and C4-M1, electrooculogram, chin electromyogram, electrocardiogram, nasal cannula, thoracic and abdominal respiratory effort, finger pulse oximetry, leg electromyogram and position sensor. Embla Titanium (Milwaukee, WI, USA), Sandman (Natus, CA, USA), Alice-5 (Philips Respironics, Murrysville, PA, USA) or Grael (Compumedics, Abbotsford, VIC, Australia) PSG systems were used. EEG was sampled at 512 Hz (Embla Titanium, Sandman), 256 Hz (Siesta) or 200 Hz (Alice-5). PSG recordings were exported to standardized European Data Format prior to quantitative EEG analyses.

Registered sleep technologists performed all sleep scoring according to standardized criteria.^43^ For descriptive purposes, sleep macro-architecture variables were reported including total sleep time (minutes), sleep efficiency (SE; the proportion of time spent asleep relative to time spent in bed, %), duration of non-rapid eye movement sleep (NREM; minutes), wake after sleep onset (amount of time spent awake after the first epoch of sleep, minutes); apnoea–hypopnoea index (number of apnoea and hypopnoea events per hour of sleep) and oxygen desaturation index (number of desaturation events with at least a 3% drop in blood oxygen).

### Quantitative EEG analysis

Overnight EEG recordings underwent automated artefact processing using previously validated detection parameters.^44^ Artefact-free epochs were analysed using a standard fast Fourier transform routine with a rectangular window applied to each non-overlapping 5-s segment for each EEG derivation. Absolute power spectra (µV^2^) were obtained in the slow oscillation frequency range (0.25–1 Hz). For each 30-s epoch, EEG power was calculated as the mean of all available artefact-free 5-s segments (up to six segments). Stage-level NREM (N2 and N3) power was then computed as the average of all 30-s epochs within the same sleep stage.

Spindle events were identified using an automated detection tool and previously validated in MCI and older adults.^12^ A 128-order band-passing Finite-Impulse-Response filter was applied to the raw EEG signal to yield a time course of sigma frequency activity (11–16 Hz). Spindle events were detected using a relative amplitude threshold approach applied to the 11–16 Hz (sigma band) envelope. Detection thresholds were defined as the median amplitude of the sigma-band signal plus its standard deviation, calculated independently for each EEG derivation to account for inter-individual and channel-specific differences in signal amplitude. Spindle events were required to meet the duration criteria of ≥ 0.5 ≤ 3.0 s and must have occurred in NREM sleep (N2 and N3). Spindle measures extracted for analysis were overall spindle density (11–16 Hz; events per minute of NREM sleep), slow spindle density (11–13 Hz; events per minute), fast spindle density (13–16 Hz, events per minute) and spindle amplitude (mean amplitude of overall spindle events *µ*V). To confirm that the detection algorithm adequately separated slow and fast spindles, spindle frequency distributions were inspected in a subset of four randomly selected participants and are presented in Supplementary Fig. 1.

All recordings were visually reviewed after power spectral analysis and spindle detection to assess signal quality and residual artefact across the night. Channels with substantial residual artefact were excluded. Slow oscillation activity and spindle event values exceeding 1.5 standard deviations from the sample mean were also visually re-checked using frequency-specific digital filters.

Sleep EEG measures were averaged across frontal (Fz, F3, and F4) and central (Cz, C3, and C4) derivations for statistical analysis. Slow oscillation power (log-transformed) and sleep spindle density were compared between the four sleep systems. Slow oscillation power was higher in the Grael system, compared to the non-Grael systems (*P* < 0.05). See Supplementary Fig. 2 for more detail. There were no differences on spindle density between the four sleep systems. See Supplementary Fig. 3 for more detail.

### MRI acquisition

All MRI was performed at the Brain and Mind Centre, University of Sydney, on a 3T GE Discovery MR750 scanner (GE Medical Systems, Milwaukee, WI, USA) with an 8 channel phased array head coil. Diffusion-weighted imaging (DWI) was collected with repetition time = 8000 ms, echo time = 84 ms, 64 slices of 2.5 mm thickness, 1.0 × 1.0 × 2.5 mm voxels, and 32 gradient directions (*b* = 1000 s/mm^2^), and two images without diffusion-weighting (*b* = 0 s/mm^2^). T1-weighted structural images were also acquired for all participants during the same scan session using a magnetization prepared rapid gradient-echo sequence with 196 sagittal slices, repetition time = 7.3 ms, echo time = 2.8 ms, flip angle = 12°, acquisition matrix = 256 × 256 with 0.9 mm isotropic voxels. On average, the MRI was performed 20.8 days from the sleep study.

### Diffusion weighted image pre-processing

**Figure 1:**
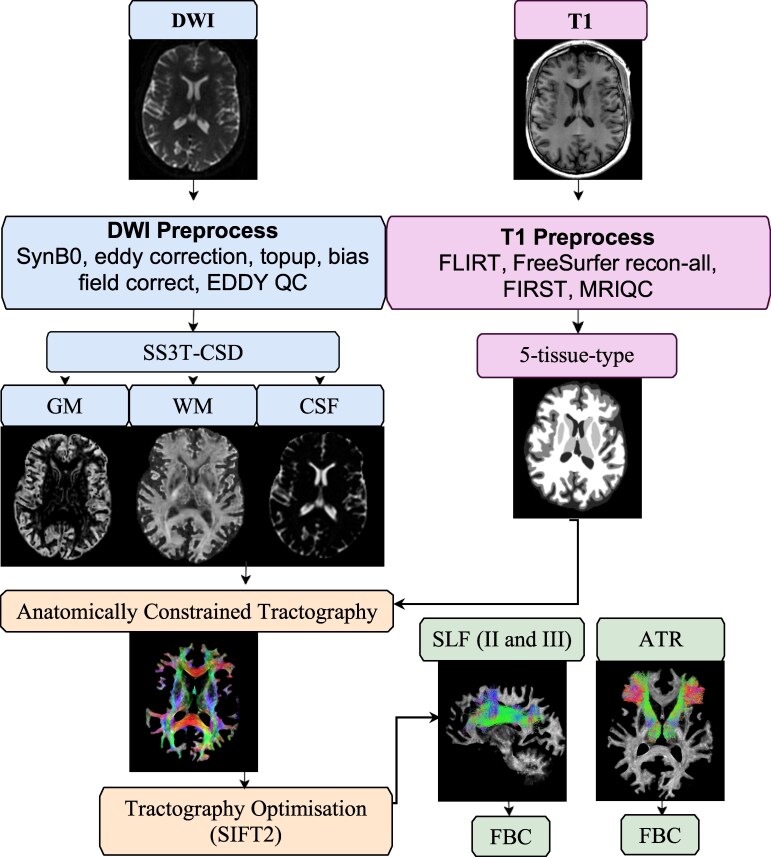
**Processing pipeline to derive FBC for each participant as described in the Methods section.** Whole brain tractogram with 10 million streamlines shown as directionally-encoded colour tract-density image (left bottom row; Calamante *et al*. ^45^). Streamlines selected from whole brain tractogram for each tract of interest displayed on tract-density image (right bottom row). All images were generated from a single participant.. Colours represent local fibre orientation (red = left–right, blue = inferior–superior, green = anterior–posterior). DWI, diffusion-weighted imaging; SS3T-CSD, single-shell 3-tissue constrained spherical deconvolution; GM, grey matter; WM, white matter; CSF, cerebrospinal fluid; SIFT2, spherical-deconvolution informed filtering of tractograms, version 2; SLF, superior longitudinal fasciculus; ATR, anterior thalamic radiation; FBC, fibre bundle capacity.

The DWI processing pipeline is depicted in Fig. 1. As reverse phase-encoded scans were not included in the diffusion-weighted acquisition, the SynB0 pipeline^46^ was used to generate a synthetic undistorted b0 image from each participant’s original distorted *b* = 0 s/mm^2^ image, T1-weighted scan and brain mask computed from the diffusion-weighted image with HD-BET.^47^ The distorted and undistorted b0 images were then used by *topup*^48^ as implemented in FSL (Version 6.0.1^49^) to correct for susceptibility induced distortions, followed by eddy current correction with FSL’s *eddy*^50^ with outlier replacement.^51^ All diffusion-weighted images were bias field corrected with advanced normalization tools N4 bias field correction^52^ and upsampled to 1.25 mm isotropic voxel size. Participant and group-level quality control of all DWI images was completed with *EDDY QC*^53^ as implemented in FSL and visual inspection.

### Structural image pre-processing

As shown in Fig. 1, each participant’s T1-weighted structural image was linearly registered to their DWI image with FSL’s *FLIRT*^54^ before being processed with the FreeSurfer *recon-all* pipeline (Version 7.1.0).^55^ A so-called five-tissue-type segmented image was then generated from the T1-weighted structural image by combining cortical surface reconstructions from FreeSurfer *recon-all* and volume based subcortical segmentations from FSL *FIRST Hybrid Surface Volume Segmentation implemented in MRtrix version 3.0.1*^56^; this segmented image was used for anatomical-constrained tractography. All MRI scans were visually inspected for quality control.

### Tractography

Group average white matter, grey matter and cerebrospinal fluid response functions were modelled by three-tissue constrained spherical deconvolution of single-shell diffusion data using MR-trix3Tissue (https://3Tissue.github.io; version 5.2.7), a fork of MRtrix3,^57^ to estimate the white matter fibre orientation distribution (FOD) for each participant. Joint bias field correction and global intensity normalization was applied to the resulting white matter FODs.^58^ Whole brain tractograms with 10 million streamlines were generated by probabilistic tractography using dynamic seeding and anatomically-constrained tractography.^59^ Streamlines were cropped at the grey matter-white matter interface with the resulting tractogram optimized through spherical-deconvolution informed filtering (SIFT2^60^, which assigns a *sift2* weight factor to each streamline in the tractogram).

Tract masks generated by TractSeg Version 2.1.1^61^ were used for the selection of streamlines from the whole brain tractogram belonging to the ATR and the SLF (portions II and III). Streamlines were discarded if they left the tract mask or did not pass through the tract endings segmentations. The cingulum was also included as an exclusionary mask, given the overlap between the ATR and anterior portion of the cingulum and that the cingulum runs parallel to the SLF bundle.

### Fibre bundle capacity

Finally, FBC was computed for each tract of interest in each participant. FBC is a quantitative measure of the connection density of a given white matter bundle.^34^ Briefly, FBC is computed as the product of a subject-specific proportionality coefficient (which matches the total track density from the tractogram to the total fibre density from the FODs) and the sum of the *sift2* weights for all the streamlines within a fibre bundle of interest. Scaling the sum of streamline weights by a proportionality coefficient, along with the use of global intensity normalization and group average response functions, allows for the unbiased inter-subject comparison of FBC.^31^ Given the absence of a priori hypotheses regarding lateralization, left and right tract measures were averaged for all analyses.

### Statistical analysis

All statistical analyses were conducted in R (version 4.4.2). Group differences between SCD and MCI groups were assessed via independent Student’s *t*-test and Mann–Whitney U tests where applicable.

The primary quantitative EEG measures of interest were slow oscillation power, and fast and slow spindle density. Additionally, overall spindle density and spindle amplitude were reported for descriptive purposes and exploratory analyses were conducted on these variables. Associations between quantitative EEG measures and FBC in each tract of interest were assessed through linear regression. Sleep micro-architecture measures were treated as outcome variables, with FBC tracts of interest as the predictors, along with age and sex being included as covariates in all models. To account for the potential moderating effects of cognitive classification (SCD and MCI), interactions between FBC and cognitive classification were included in all models. Main effects models were interpreted where an interaction was not statistically significant. When the interaction effect was statistically significant, a simple slope analysis was conducted to investigate the association between the EEG measure of interest and the tract of interest within the SCD and MCI groups, whilst adjusting for age and sex. Effect sizes were estimated using partial *R*^2^, calculated as the proportion of variance in the dependent variable uniquely explained by each predictor while controlling for other covariates in the model.

We conducted exploratory analyses to characterize interrelationships among slow and fast spindles, as well as white matter tracts. These analyses were intended to contextualize the main tract-spindle associations and to inform interpretation regarding white matter alteration specificity.

For non-normally distributed data, appropriate transformations were applied to the demographic and sleep micro-architecture prior to data analysis. To control for multiple comparisons, false discovery rate (FDR) corrections using the Benjamini–Hochberg procedure were applied. Families of tests were defined based on distinct outcomes and hypotheses. Main effects and interaction effects were treated as separate families, as they address different scientific questions (i.e. overall spindle-white matter associations versus moderation by cognitive diagnosis). Within each family, FDR correction was applied across parallel tests conducted across predefined white matter tracts. For example, a single family comprised the associations between frontal and central fast sleep spindles and the ATR, SLF-II and SLF-III (two spindle metrics × three tracts; six tests in total). Post hoc simple slope analyses were treated as a separate family of tests and corrected independently using the same Benjamini–Hochberg procedure. Adjusted *P*-values are reported as *q*-values, with statistical significance defined as *q* < 0.10.

Lastly, to assess the robustness of the findings, sensitivity analyses were conducted by excluding outliers in spindle amplitude. Outliers were defined as values exceeding ±3 SD from the mean within each region. Two outliers in the frontal region and two in the central region were removed, and significant regression analyses were re-run.

## Results

A total of 67 participants met the eligibility criteria for this sub-study (mean age = 68.1, SD = 8.2). Of these participants, 21 were classified as SCD and 46 as MCI. SCD and MCI groups differed in MMSE and BMI (*P* < 0.05). There were no other group differences in clinical demographics (see Table 1 for all group comparisons in clinical demographics). There were no differences in the tracts of interest between the SCD and MCI groups, see Supplementary Table 1.

**Table 1:** Clinical summary statistics by cognitive classification

	SCD *n* = 21	MCI *n* = 46	Test statistic	*P*-value
Age (years)	65.3 (6.1)	69.4 (8.7)	−1.98	0.052
Sex, female (%)^a^	16 (84.2%)	31 (65.0%)	1.40	0.237
WTAR	107.0 (10.8)	110 (0.9)	246	0.495
MMSE,/30^b,c^	30.0 (1.0)	29.0 (1.8)	**632.5**	**0**.**013**
GDS-15,/15^c,d^	4.2 (3.2)	3.5 (3.8)	1.33	0.188
BMI	24.5 (4.1)	27.9 (5.9)	**−2**.**28**	**0**.**026**
CIRS-G^c,d^	3.0 (2.5)	4.5 (4.0)	−1.58	0.168
PSQI total score,/21^c,e^	8.4 (3.5)	7.1 (3.6)	1.48	0.145
Antidepressants (%)^1^	4 (26.7%)	6 (16.2%)	0.05	0.826

Independent student’s *t*-test were conducted unless otherwise noted.

^a^Chi-square test; mean (SD) are presented.

^b^Mann–Whitney *U* test was conducted.

^c^Median (interquartile range) was reported.

^d^Log transformation was performed prior to conducting independent student’s *t*-test.

^e^Square root transformation was performed prior to conducting independent student’s *t*-test. Group differences are shown in bold; GDS-15, Geriatric Depression Scale–15 item; BMI, body mass index; CIRS-G, Cumulative Illness Rating Scale-Geriatric version; PSQI, Pittsburgh Sleep Quality Index.

### Group differences in sleep macro and micro-architecture

Sleep summary statistics are presented in Table 2. The SCD and MCI groups did not differ in their sleep macro-architecture measures of total sleep time, sleep efficiency, amount of time spent awake, NREM sleep duration or sleep apnoea severity measures (apnoea–hypopnoea index, oxygen desaturation index).

**Table 2:** Sleep summary statistics by cognitive classification

	SCD *n* = 21	MCI *n* = 46	Test statistic	*P*-value
*Polysomnography*				
TST (mins)	341.4 (68.6)	343.5 (61.7)	−0.12	0.906
NREM (mins)	283.3 (68.2)	286.9 (46.6)	−0.23	0.818
REM (mins)	60.5 (27.4)	60.1 (27.0)	0.05	0.958
WASO (mins)^a,b^	57.5 (45.6)	74.5 (50.8)	−1.06	0.293
SE (%)^a,c^	79.3 (19.4)	78.6 (11.0)	0.09	0.925
AHI^a,b^	9.5 (21.6)	13.2 (14.9)	−1.09	0.281
ODI^a,b^	7.7 (12.1)	7.4 (13.7)	−0.89	0.375
*Slow-wave power*				
Frontal^a,b^	175.7 (109.5)	194.2 (172.1)	0.23	0.821
Central^a,b^	184.8 (112.1)	157.3 (112.0)	0.43	0.341
*Total spindle density*				
Frontal^a,b^	1.3 (1.5)	0.7 (1.0)	**4**.**09**	**0**.**001**
Central^a,b^	1.2 (0.5)	0.5 (0.9)	**3**.**94**	**0**.**001**
*Fast spindle density*				
Frontal^a,b^	0.3 (0.3)	0.05 (0.1)	**3**.**63**	**0**.**001**
Central^a,b^ *Slow spindle density*	0.7 (0.4)	0.2 (0.4)	**3**.**16**	**0**.**002**
Frontal^a,b^	0.9 (0.8)	0.6 (0.8)	**2**.**72**	**0**.**008**
Central^a,b^ *Spindle amplitude*	0.5 (0.4)	0.3 (0.5)	1.68	0.098
Frontal^a,b^	15.3 (5.2)	15.9 (5.7)	−0.66	0.514
Central^a,b^	16.2 (3.9)	16.4 (7.4)	−0.71	0.482

Independent student’s *t*-test were conducted and Mean (SD) were reported unless otherwise noted.

^a^Median (interquartile range) was reported.

^b^Log transformation was applied prior to independent student’s *t*-test.

^c^Square transformed was applied prior to independent student’s *t*-test. Group differences are shown in bold. TST, total sleep time; WASO, wake after sleep onset; SE, sleep efficiency; spindle density = events per minute; spindle amplitude = mean amplitude during NREM.

During NREM sleep, the MCI group had lower overall and fast spindle densities during NREM sleep in the frontal and central brain regions. In addition, a lower slow spindle density in the frontal region was observed for the MCI group compared to the SCD group. There were no between group differences in slow oscillation power or mean spindle amplitude.

### White matter connectivity and sleep micro-architecture

#### Slow spindle density

There were no statistically significant associations between the FBC of the ATR, SLF-II, and SLF-III and slow spindle density in the frontal and central regions (Table 3).

**Table 3:** Slow spindle density linear regression summary

		Frontal (*n* = 67)				Central (*n* = 67)			
		*Β*	Partial $R^{2}$	*P*-value	*q*-value	β	Partial $R^{2}$	*P*-value	*q*-value
ATR	Main effect	−0.22	0.00	0.291	0.582	−0.28	0.00	0.202	0.582
	Int	0.21	0.01	0.408	0.601	0.35	0.03	0.190	0.570
SLF-II	Main effect	0.18	0.00	0.419	0.629	−0.01	0.00	0.961	0.961
	Int	−0.34	0.03	0.186	0.570	−0.18	0.01	0.501	0.601
SLF-III	Main effect	0.09	0.00	0.629	0.755	−0.24	0.00	0.213	0.582
	Int	0.04	0.00	0.883	0.883	0.22	0.01	0.381	0.601

To control for multiple comparisons, false discovery rate correction was applied separately to main effects and tract × diagnosis interaction effects. Within each effect family, FDR correction was applied across the six parallel tests using the Benjamini–Hochberg procedure. Adjusted *P*-values are reported as *q*-values, and statistical significance was defined as *q* < 0.10. Simple slopes analysis of significant interaction effects is reported in text. Int, interaction between FBC and cognitive classification.

#### Fast spindle density

**Figure 2:**
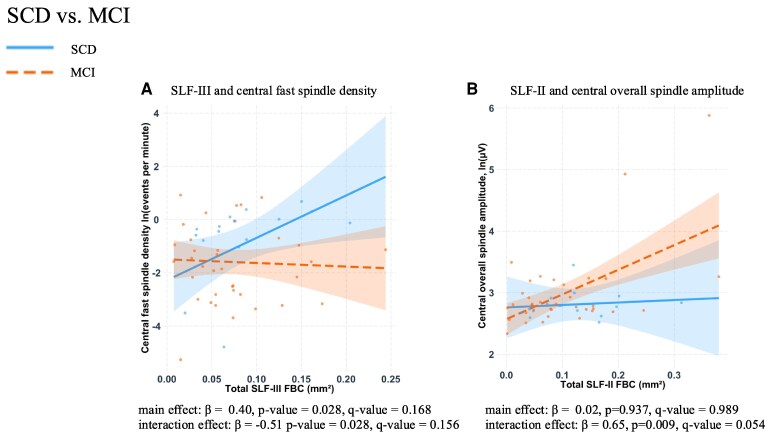
**Relationship between sleep spindle characteristics and superior longitudinal fasciculus (SLF-II and SLF-III) within clinical groups.** (**A**) Within-group associations between fast spindle density and fibre bundle capacity of the SLF-III. (**B**) Within-group associations between spindle amplitude and fibre bundle capacity of the SLF-II. The interaction effect between spindle amplitude and fibre bundle capacity of the SLF-II was significant after FDR correction, and the relationship was driven by the MCI group. Associations were tested using linear regression models with the dependent variable as the spindle metric and fibre bundle capacity with cognitive status (SCD versus MCI) as an interaction term. Total sample size was *N* = 67 (SCD *n* = 21; MCI *n* = 46). Each data point represents one participant’s raw value. Abbreviations: SCD = subjective cognitive decline; MCI = mild cognitive impairment; SLF = superior longitudinal fasciculus.

As presented in Table 4, there was a significant main and interaction effect between increased central fast spindle density and SLF-III; however, this did not survive FDR corrections (*q* > 0.10). The interaction effect explained 8% unique variance (partial R^2^). See Fig. 2A for the within-group scatterplot between central fast spindle density and SLF-III. There were no other significant associations between fast spindles and any other tracts before or after FDR corrections.

**Table 4:** Fast spindle density linear regression summary

		Frontal (*n* = 67)				Central (*n* = 67)			
		β	Partial $R^{2}$	*P*-value	*q*-value	β	Partial $R^{2}$	*P*-value	q-value
ATR	Main effect	0.24	0.00	0.236	0.472	0.05	0.00	0.809	0.809
	Int	−0.17	0.01	0.490	0.791	−0.11	0.00	0.659	0.791
SLF-II	Main effect	−0.08	0.00	0.696	0.809	0.31	0.06	0.149	0.447
	Int	0.12	0.00	0.642	0.791	−0.49	0.06	0.052	0.156
SLF-III	Main effect	0.06	0.00	0.745	0.809	0.40	0.00	0.028	0.168
	Int	−0.03	0.00	0.904	0.904	−0.51	0.08	0.028	0.156

To control for multiple comparisons, false discovery rate correction was applied separately to main effects and tract × diagnosis interaction effects. Within each effect family, FDR correction was applied across the six parallel tests using the Benjamini–Hochberg procedure. Adjusted *P*-values are reported as *q*-values, and statistical significance was defined as *q* < 0.10. Simple slopes analysis of significant interaction effects is reported in text. Int, interaction between FBC and cognitive classification.

#### Slow oscillatory power

There were no significant main effects of slow oscillatory power and the FBC of the ATR, SLF-II and SLF-III for both frontal and central regions (Table 5). Similarly, there were no significant interaction effects between slow oscillatory power and the tracts of interest. See Table 5 for each model’s estimate. Given slow oscillatory power was different between Grael and non-Grael sleep systems, it was included as a covariate in a subsequent sensitivity analysis (see Supplementary Table 2). Inclusion of sleep systems did not alter the results.

**Table 5:** Slow oscillatory power linear regression summary

		Frontal (*n* = 61)				Central (*n* = 65)			
		β	Partial $R^{2}$	*P*-value	*q*-value	β	Partial $R^{2}$	*P*-value	*q*-value
ATR	Main effect	−0.07	0.00	0.751	0.774	−0.06	0.00	0.771	0.774
	Int	0.02	0.00	0.928	0.928	−0.01	0.00	0.975	0.925
SLF-II	Main effect	−0.07	0.00	0.774	0.774	−0.22	0.00	0.324	0.774
	Int	0.272	0.02	0.322	0.486	0.24	0.01	0.359	0.486
SLF-III	Main effect	−0.144	0.00	0.432	0.774	−0.25	0.00	0.158	0.774
	Int	0.469	0.06	0.073	0.438	0.47	0.06	0.063	0.474

To control for multiple comparisons, false discovery rate correction was applied separately to main effects and tract × diagnosis interaction effects. Within each effect family, FDR correction was applied across the six parallel tests using the Benjamini–Hochberg procedure. Adjusted *P*-values are reported as *q*-values, and statistical significance was defined as q < 0.10. Simple slopes analysis of significant interaction effects is reported in text. Int, interaction between FBC and cognitive classification.

### Exploratory analyses

#### Overall sleep spindle density

There were no associations between overall sleep spindle density and the FBC of the tracts of interest (see Supplementary Table 3).

#### Spindle amplitude

The overall main effects of ATR, SLF-II and SLF-III were not associated with spindle amplitude. However, there was a significant interaction between SLF-II and cognitive classification on spindle amplitude for both the frontal and central regions (Table 6). The interaction effect remained significant after FDR corrections, and adjustment for age and sex, and explained 7% (frontal) and 11% (central) unique variance. Simple slope analyses revealed that, in individuals with MCI, higher FBC in the SLF-II was associated with greater spindle amplitude in frontal regions (slope estimate = 7.65, *t* = 5.2, *P* = 0.001, *q* = 0.001) and central regions (slope estimate = 4.0, *t* = 4.3, *P* = 0.001, *q* = 0.001), after controlling for age and sex. While for the SCD group, none of the slope estimates reached statistical significance (both *P* > 0.05 and *q* = 0.937) across the significant interaction analyses. See Fig. 2B for within-group scatterplots.

**Table 6:** Spindle amplitude linear regression summary

		Frontal (*n* = 67)				Central (*n* = 67)			
		β	Partial $R^{2}$	*P*-value	*q*-value	β	Partial $R^{2}$	*P*-value	*q*-value
ATR	Main effect	0.04	0.00	0.871	0.989	0.06	0.00	0.813	0.989
	Int	0.17	0.01	0.555	0.555	0.17	0.01	0.554	0.555
SLF-II	Main effect	0.03	0.01	0.872	0.989	0.02	0.00	0.937	0.989
	Int	**0**.**57**	**0**.**07**	**0**.**026**	**0**.**078**	**0**.**65**	**0**.**11**	**0**.**009**	**0**.**054**
SLF-III	Main effect	−0.02	0.00	0.929	0.989	0.01	0.00	0.989	0.989
	Int	0.20	0.01	0.461	0.555	0.25	0.01	0.351	0.555

To control for multiple comparisons, false discovery rate correction was applied separately to main effects and tract × diagnosis interaction effects. Within each effect family, FDR correction was applied across the six parallel tests using the Benjamini–Hochberg procedure. Adjusted *P*-values are reported as *q*-values. Bold text indicates statistical significance at *q* < 0.10. Simple slopes analysis of significant interaction effects is reported in text. Int, interaction between FBC and cognitive classification.

Sensitivity analyses excluding two outliers (defined as values exceeding ±3 standard deviations from the mean) yielded comparable results for the central derivation. In the MCI group, higher SLF-II FBC values remained significantly associated with greater central spindle amplitude (β = 1.96, *t* = 3.43, *P* < 0.01), consistent with the primary analysis. However, in the frontal derivation, exclusion of two outliers attenuated this association, such that SLF-II FBC was no longer a significant predictor of frontal spindle amplitude in the MCI group (β = 0.79, *t* = 0.92, *P* = 0.365).

For the frontal region, the densities of fast and slow spindles were moderately correlated (*r* = 0.38, *P* = 0.001). However, central fast and slow spindles were not correlated (*r* = 0.16, *P* > 0.05). For the tract measures, there was a small correlation between the ATR and SLF-II (*r* = 0.26, *P* = 0.03) and SLF-III (*r* = 0.27, *P* = 0.03). There was a strong correlation between SLF-II and SLF-III (*r* = 0.61, *P* = 0.001).

## Discussion

This novel study examined how sleep spindles and slow wave oscillations relate to the structural connectivity of key white matter tracts in older adults at risk for dementia, attending a brain health and memory clinic and with either subjective or mild cognitive decline. Our initial hypothesis that greater sleep spindles are associated with increased ATR, SLF-II and SLF-III connectivity was partially supported. Although significant main and interaction effects were initially found between central fast spindles and SLF-III, these associations did not survive correction for multiple comparisons using FDR, suggesting that the findings should be interpreted with caution. In our exploratory analyses, we observed that spindle amplitude was linked to SLF-II, particularly in those with mild cognitive impairment. There were no associations between slow spindle density or slow oscillatory power with any of the tracts of interest.

Although these associations did not survive FDR correction, the observed pattern linking central fast spindle activity to SLF-III and SLF-II remains of interest. Notably, the interaction between SLF-III and cognitive status accounted for 8% of the variance, while the main effect of SLF-II explained 6%. his pattern is consistent with prior evidence implicating SLF-III in cortico-cortical communication between frontal and parietal regions, supporting the coordination of fast spindle oscillations within central regions.^62^ Furthermore, our exploratory analyses demonstrated that for individuals with MCI, greater SLF-II connectivity was associated with higher spindle amplitude, a relationship that was not observed in the SCD group. One potential explanation for this finding is a shift in the large-scale neural networks recruited to support spindle expression in individuals with MCI. Spindle density and amplitude are partially distinct physiological processes. Spindle density may be more reflective of the probability of spindle initiation within the thalamo-reticular circuitry, whereas amplitude reflects the magnitude of synchronous cortical engagement once a spindle is expressed. Therefore, it may explain why the SLF is linked more strongly to spindle amplitude than spindle density. In the context of MCI, where white matter changes^63^ and altered spindle dynamics^17^ are present, this association may reflect increased dependence of large-scale network synchrony. Alternatively, the findings may reflect stable, trait-like covariance whereby individuals with greater white matter integrity exhibit consistently higher spindle amplitude independent of disease-related change, indicating pre-existing individual differences in neurobiological capacity rather than a compensatory process. Unfortunately, the cross-sectional nature of this study cannot determine longitudinal changes; further work examining relationships between larger-scale brain networks and sleep micro-architecture may provide valuable insights. Notably, the SLF-II and SLF-III FBC metrics were intercorrelated, consistent with shared variance across the adjacent frontoparietal pathways. In contrast, there was only a small correlation between the two SLF branches and ATR. This dissociation suggests that the observed associations with spindle activity are anatomically specific rather than reflective of global or non-specific white matter alterations.

Our results did not support an association between ATR connectivity and slow oscillatory power. Slow-wave oscillations are cortically generated to promote the downscaling of neuronal synapses, preventing oversaturation of synaptic strength and conserving energy.^8^ A smaller body of emerging literature has also shown age-related changes in sleep micro-architecture, including reductions in slow-wave activity during NREM sleep,^64^ but limited evidence was found for associations between white matter connectivity and slow-wave power. Slow-wave oscillations have been suggested to rely on effective cortico-cortical connectivity^27^ and found to be associated with white matter hyperintensities^65^ and micro-structural white matter properties in healthy young adults.^66^ However, another previous study^66^ assessed the steepness of the rising slope of slow-wave oscillations, which is thought to represent the synchronization of the transition from hyperpolarization to depolarization.^67^ While here, we examined slow oscillatory power, it is possible that the relationship between white matter properties and slow oscillations may be specific to other features of slow-wave activity, such as the rising slope of oscillations or the number of slow oscillatory events. Alternatively, slow oscillation propagation could be reliant on short-range cortico-cortical connections rather than connectivity of longer-range major white matter fibre bundles such as those assessed here.

In terms of clinical implications, the primary findings of associations between white matter connectivity and sleep spindles may have implications for cognitive functioning in older adults. For example, one study^26^ found white matter alterations to be associated with lower spindle density and poorer overnight motor memory consolidation in older adults, with spindles also implicated in declarative memory consolidation (see review^10^). Given white matter properties have been implicated in cognitive decline,^24^ it is possible that white matter represents a common mechanism linking subtle cognitive changes, potentially related to sleep micro-architecture mediated reductions in overnight consolidation and progression to more severe, objectively measurable cognitive impairments. Moreover, recent evidence linking CSF markers of neurodegeneration to sleep spindle activity^68^ suggests that longitudinal studies aimed at disentangling the contributions of white matter and neuropathology to overnight memory consolidation and cognitive functioning are required.

There are some limitations of this work that should be considered. First, the optimal methods for the selection of streamlines from whole-brain tractography remain an open problem. Similarly, the diffusion sequence used in this study uses a relatively lower angular resolution (32 diffusion directions) which may affect fibre orientation estimation and tractography precision. However, the reconstruction and inclusion/exclusion masking methods used in this study contributed to minimize spurious streamlines. Second, although the cognitive classifications were made based on detailed clinical and neuropsychological assessments, we were not able to assess participants’ underlying pathological burden with established biomarkers. Future work investigating white matter connectivity and sleep micro-architecture in the context of neurodegenerative pathology is warranted. Third, although slow oscillation power was examined, we did not perform event-based slow oscillation detection or slow oscillation–spindle coupling analyses. These measures may provide additional insight into sleep-dependent memory mechanisms in ageing and cognitive impairment and warrant investigation in future studies. Fourth, our sample size was relatively small, particularly for within-group associations, together with the cross-sectional design, limits our ability to draw conclusions about directionality or change over time. Fifth, we did not control daytime experiences or prior-night sleep, which could alter slow oscillatory power through differences in sleep pressure or daytime experience (e.g. novel learning), which may affect spindle activity at night. Effects of this were likely mitigated through the use of single-shell three-tissue constrained spherical deconvolution, meaning the grey matter-like and CSF-like signal within regions of white matter lesions^69^ was modelled and excluded, with fibre tracking based on the white matter-like signal only. Lastly, we did not control for sleep apnoea and depressive symptoms. While it is possible that other factors such as sleep disorders and depressive symptoms influenced our findings, it is noted that we excluded those taking hypnotics, sedatives, mood stabilizers, anti-epileptics, anti-psychotics, benzodiazepines, and cholinesterase inhibitors. Furthermore, depressive symptoms were relatively low, and SCD and MCI groups did not differ in sleep apnoea severity or depressive symptoms

In this study, we identified for the first-time relationships between overnight sleep micro-architecture and white matter connectivity of select white matter tracts in older adults at risk for cognitive decline, and showed that cognitive classification moderated relationships between sleep spindle amplitude and white matter connectivity. These findings highlight a number of important considerations for future work aiming to understand complex interactions between sleep micro-architecture and neurobiological factors.

## Supplementary Material

fcag303_Supplementary_Data

## Data Availability

Due to ethical considerations, data used in this article will be shared upon reasonable request to the corresponding author. The code supporting the findings of this study is available at: osf.io/uy7jk.
