# Supplementary material for: Sleep spindles are linked to white matter connectivity differentially in older adults with and without cognitive impairment
Source: Brain Commun. 2026 Aug 10;8(4):fcag303. doi: 10.1093/braincomms/fcag303 (PMC13458153; doi:10.1093/braincomms/fcag303)
Supplement: Supplementary file 1 [file fcag303_supplementary_data.docx]

| Supplementary Table 1. Differences in tracts of interest between SCD and MCI groups | | | | |
| --- | --- | --- | --- | --- |
|  | SCD  n = 21 | MCI  N = 46 | Test statistic | p-value |
| Total ATR | 0.41 (0.44) | 0.18 (0.55) | 588 | 0.159 |
| Total SLF-II | 0.12 (0.15) | 0.09 (0.10) | 571 | 0.239 |
| Total SLF-III | 0.08 (0.07) | 0.06 (0.06) | 618 | 0.069 |
| *p<0.05. Statistically significant group differences are indicated in bold. Mann-Whitney U test were conducted. Data is presented as median (interquartile range). SCD = subjective cognitive decline; MCI = mild cognitive impairment; ATR = anterior thalamic radiation; SLF = superior longitudinal fasciculus | | | | |

| Supplementary Table 2. Slow oscillatory power linear regression sensitivity analysis including PSG system as a covariate | | | | | |
| --- | --- | --- | --- | --- | --- |
|  | | Frontal (n=61) | | Central (n=65) | |
|  | | β | p-value | β | p-value |
| ATR | Main effect | -0.11 | 0.624 | -0.05 | 0.864 |
|  | Int | -0.06 | 0.830 | -0.01 | 0.965 |
| SLF-II | Main effect | 0.01 | 0.972 | -0.03 | 0.909 |
|  | Int | 0.05 | 0.853 | 0.07 | 0.814 |
| SLF-III | Main effect | -0.09 | 0.651 | -0.12 | 0.569 |
|  | Int | 0.21 | 0.425 | 0.30 | 0.296 |
| Note:Int = interaction between FBC and cognitive classification. PSG = polysomnography; ATR = anterior thalamic radiation; SLF = superior longitudinal fasciculus; Int = interaction between FBC and cognitive classification. | | | | | |

| Supplementary Table 3. Overall spindle density linear regression summary | | | | | | | | | |
| --- | --- | --- | --- | --- | --- | --- | --- | --- | --- |
|  | | Frontal (n=67) | | | | Central (n=67) | | | |
|  | | β | Partial $R^{2}$ | p-value | q-value | β | Partial $R^{2}$ | p-value | q-value |
| ATR | Main effect | -0.09 | 0.00 | 0.639 | 0.664 | -0.09 | 0.00 | 0.664 | 0.664 |
|  | Int | 0.07 | 0.00 | 0.755 | 0.841 | -0.05 | 0.00 | 0.841 | 0.841 |
| SLF-II | Main effect | 0.09 | 0.00 | 0.655 | 0.664 | 0.09 | 0.00 | 0.652 | 0.664 |
|  | Int | -0.26 | 0.02 | 0.285 | 0.841 | -0.32 | 0.03 | 0.181 | 0.841 |
| SLF-III | Main effect | -0.09 | 0.00 | 0.620 | 0.664 | 0.08 | 0.00 | 0.644 | 0.664 |
|  | Int | -0.05 | 0.00 | 0.819 | 0.841 | -0.12 | 0.00 | 0.594 | 0.841 |
| Note: Int = interaction between FBC and cognitive classification. ATR = anterior thalamic radiation; SLF = superior longitudinal fasciculus; Int = interaction between FBC and cognitive classification. | | | | | | | | | |

**Supplementary Figure 1. Frequency distribution of sleep spindles across C3 and F4 electrodes for four randomly selected participants.**

Each panel represents an individual participant. Frequency histograms are shown separately for frontal (F3) and central (C3) electrodes. A nominal boundary of 13 Hz is indicated to illustrate slow and fast spindle ranges.

A) Subject 1: A high prevalence of slow spindles was observed at the frontal electrode, whereas central recordings demonstrated a bimodal distribution of both slow and fast spindles.

B) Subject 2: A high prevalence of slow spindles was observed at the frontal electrode, whilst a high number of slow spindles were detected at the central electrode.

C) Subject 3: A similar pattern to subject 2, with a high number of frontal slow spindles and central fast spindles.

D) Subject 4: Frequency distributions show a weaker bimodal structure across both electrodes, indicating a poor separation between slow and fast spindles.

**Supplementary Figure 2. Difference between slow oscillation power between PSG systems**

Polysomnography (PSG) system was significantly associated with log absolute NREM slow-wave power in both frontal and central regions. (A) For central power (n=65), there was also a significant overall effect, *F*(3, 61) = 5.25, *p* = .003. Tukey-adjusted comparisons showed lower central power for REMlogic and Sandman compared with Grael (p<0.05); Alice did not differ significantly from Grael or the other systems (p>0.05). (B) For frontal power (n=61), there was a significant overall effect of PSG system, *F*(3, 57) = 9.35, *p* < .001. Tukey-adjusted comparisons showed lower frontal power for Alice, REMlogic, and Sandman compared with Grael (p<0.05), while the non-Grael systems did not differ from each other (p>0.05). Each dot represents an individual participant. NREM = non-rapid eye movement; PSG = polysomnography.

**Supplementary Figure 3. Difference between spindle density between PSG systems**

Polysomnography (PSG) system was not significantly associated with log NREM spindle density in either frontal or central regions. (A) For central spindle density (n=67), the overall effect of PSG system was not significant, *F*(3, 63) = 1.98, *p* = .127, and all Tukey-adjusted pairwise comparisons were non-significant. (B) Similarly, for frontal spindle density (n=67), there was no significant overall effect of PSG system, *F*(3, 63) = 1.77, *p* = .162, and Tukey-adjusted pairwise comparisons were not significant. Each dot represents an individual participant. NREM = non-rapid eye movement; PSG = polysomnography.
